# Thyroid Functions in Long-Term Survivors of Pediatric Hodgkin’s Lymphoma Treated with Chemotherapy and Radiotherapy

**DOI:** 10.4274/jcrpe.v3i2.18

**Published:** 2011-06-08

**Authors:** Metin Demirkaya, Betül Sevinir, Halil Sağlam, Lütfi Özkan, Okan Akacı

**Affiliations:** 1 Division of Pediatric Oncology, Department of Pediatrics, Uludag University, Medical Faculty, Bursa, Turkey; 2 Division of Pediatric Endocrinology, Department of Pediatrics, Uludag University, Medical Faculty, Bursa, Turkey; 3 Department of Radiation Oncology, Uludag University, Medical Faculty, Bursa, Turkey; 4 Department of Pediatrics, Uludag University, Medical Faculty, Bursa, Turkey

**Keywords:** Hodgkin disease, children, late effect

## Abstract

**Objective:** Post-treatment endocrine disturbances are common in cancer patients who have received radiotherapy or chemotherapy. The objective of this study was to evaluate the thyroid functions of long-term survivors of pediatric Hodgkin’s lymphoma treated with chemotherapy and radiotherapy.

**Methods:** Thyroid functions of 55 Hodgkin’s lymphoma patients (M/F:2.05/1) in complete remission were evaluated retrospectively.

**Results:** The mean age of the patients at diagnosis was 10.35±4.09 (range: 2.83-17) years and the mean follow-up period was 5.54±3.68 (range: 0.92-13.92) years. All patients received chemotherapy; a total of 50 patients (90.9%) underwent radiotherapy, 42 (76.4%) of whom received neck/mantle radiotherapy. Thyroid function tests were abnormal in 14 (24.5%) patients and normal - in the remaining 41 (74.5%). A diagnosis of subclinical and overt hypothyroidism was made in 11 (78.6%) and 3 (21.4%) patients with abnormal thyroid function tests, respectively. Nearly one-fourth (21.4%) of all thyroid function disorders were detected in the first year of follow-up. A statistically significant correlation was found between the dose of mantle radiotherapy and thyroid function disorder (p=0.002). In addition, statistically significant correlations were established between thyroid examination or thyroid ultrasonography findings and thyroid functions (p <0.001 or p=0.006, respectively).

**Conclusions:** Radiation-induced thyroid disorders may develop in pediatric Hodgkin’s lymphoma patients in complete remission starting as early as the first year after treatment and are dose-dependent. Patients, particularly those who have been exposed to radiotherapy of the neck, must be followed up closely for occurrence of thyroid dysfunctions.

**Conflict of interest:**None declared.

## INTRODUCTION

**Introduction**

In parallel with enhanced survival rates of pediatric Hodgkin’s lymphoma patients by current chemotherapy and radiotherapy protocols, an increase in early and late complication rates have also been noted. Common late complications of childhood cancers after treatment include neurological, cardiac, pulmonary and gonadal disorders, reduction in bone mass, defective bone and soft tissue development, and increased susceptibility to secondary malignant tumors (1,2,3,4,5,6,7,8,9). Hyperthyroidism including Graves’ disease, thyroiditis, hypothyroidism, thyroid nodules and thyroid cancers are the most commonly encountered thyroid gland disorders that are known to be related with radiotherapy (6,10,11,12,13). The objective of this study was to evaluate thyroid function in pediatric Hodgkin’s lymphoma patients in complete remission with chemotherapy and radiotherapy.

## METHODS

**Methods**

In this retrospective study, we assessed the thyroid functions in 55 pediatric Hodgkin’s lymphoma patients in complete remission who were treated in the Department of Pediatric Oncology at Uludag University Medical Faculty between January 1995 and December 2008. Recurrence occurred only in 2 patients who received a second treatment regimen and were then followed up in complete remission. Physical and thyroid examination  findings, thyroid stimulating hormone (TSH), free triiodothyronine (fT3), free thyroxine (fT4), total T3, and total T4 levels, thyroid imaging (ultrasonography and/or scintigraphy), thyroid antibody data, serum thyroglobulin and calcitonin levels were obtained from patient records and evaluated.Age and gender of the patients, stage and histopathological subgroup of the disease, chemotherapy protocols, number of cycles, radiotherapy region and dose, and follow-up durations were noted. Patients with hypothyroidism were divided into two groups as subclinical hypothyroidism and overt hypothyroidism.

**The study was approved by the local ethics committee.**

The following four chemotherapy protocols were used: i) ABVD (adriamycin 25 mg/m2 1st and 15th days, intravenously (i.v.), bleomycin 10 mg/m2 1st and 15th days i.v., vinblastine 6 mg/m2 1st and 15th days i.v., dacarbazine 375 mg/m2 1st and 15th days i.v.); ii) COPP (cyclophosphamide 600 mg/m2 1st and 8th days i.v., vincristine 1.4 mg/m2 1st and 8th days i.v., procarbazine 100 mg/m2 1-14 days p.o., prednisolone 40 mg/m2 1-14 days p.o.), iii) MOPP (mechlorethamine 5 mg/m2 1st and 8th days i.v., vincristine 1.4 mg/m2 1st and 8th days i.v., procarbazine 100 mg/m2 1-14 days p.o., prednisolone 40 mg/m2 1-14 days p.o.), iv) MOPP/ABVD or COPP/ABVD hybrid treatment protocol. The COPP, MOPP or hybrid protocols were applied to the patients between 1995 and 1997, while ABVD treatment protocol was given after 1997. All subjects, except for those who experienced recurrence, received 3-6 chemotherapy cycles. Radiotherapy regions were grouped as: i) neck region and mantle radiotherapy ii) mediastinum, iii) other regions. The patients received a total dose of 2520 centigrays (cGy) mantle radiotherapy with a daily dose of 180 cGy. The dose of radiotherapy was completed to 3060 or 3600 in patients with remaining lymphadenopathy after standard dose chemotherapy and in those with residual disease after radiotherapy, respectively. Serum total T3, total T4, fT3, fT4 and TSH levels were analyzed by Immulite 2000 autoanalyzer using chemiluminescent microparticle immunoassay (CMIA) method (Abbott Laboratories). The statistical analyses were performed using SPSS (Statistical Package for the Social Sciences) for Windows 13.0 software. Comparisons of continuous variables in multiple groups were made applying the Kruskal-Wallis test, while comparisons between two groups were made using the Mann-Whitney U test. Inter-group differences of categorical variables were determined by the Pearson’s chi-square test and Fisher’s exact test. Results were presented as mean±standard deviation (SD) values. A p-value of less than 0.05 was considered statistically significant.

## RESULTS

Table 1 lists the demographic features of the 55 patients included in this study. The patients were classified according to their thyroid function tests as normal (group 1) and abnormal (group 2). The total study population consisted of 37 males (67.3%) and 18 females (32.3%); male/female ratio was 2.05/1. Thyroid function tests were normal in 41 (74.5%) patients, while thyroid dysfunction was present in 14 (25.5%) patients. No statistically significant difference was found with regard to thyroid dysfunction between genders. The mean ages of the patients at diagnosis and when the study was performed were 10.35±4.09 (range: 2.83-17) years and 15.84±3.91 (range: 6.91-24) years, respectively. The mean follow-up duration was 5.54±3.68 (range 0.92-13.92) years. No statistically significant difference was found with regard to gender distribution or follow-up duration between groups 1 and 2. Thyroid examination was normal (grade 0) in 40 (72.7%) patients, while grade I, grade II, and grade III goiter was detected in 9 (16.4%), 5 (9.1%), and 1 (1.8%) patient, respectively. Thyroid ultrasonography  performed in 45 (81.8%) out of 55 patients was normal in 41 (91.1%) and abnormal in 4 (8.9%) patients (nodules in 3 patients and hypoechoic cyst in 1 patient). Thyroid functions were normal in 95% of patients with normal thyroid examination findings, in 22.2% of patients with grade I goiter and in 20% of patients with grade II goiter. One patient with grade III goiter had abnormal thyroid functions. Thyroid functions were significantly correlated with thyroid examination findings (p<0.001). 

Thyroid ultrasonography was performed in 87.8% and 64.3% of subjects in group 1 and 2, respectively. Thyroid functions were normal in 85.7% of patients with normal ultrasonographic findings; in contrast, thyroid dysfunction was found in all 4 patients with abnormal ultrasonographic findings. A significant correlation was observed between thyroid 

ultrasonography findings and thyroid functions (p=0.006). The 4 patients in group 2 who showed abnormal ultrasonographic findings included 3 patients with nodules and one patient with a 12x17 mm hypoechoic cystic degeneration on the right thyroid lobe. Hypothyroidism was present in all 4 patients. Thyroglobulin levels in the case with hypoechoic cyst were higher than normal and the patient underwent thyroid surgery. The cyst was benign in nature on histopathological evaluation. As shown in Table 2, histopathological typing revealed that the Hodgkin’s lymphoma was nodular sclerosing in 28 (50.9%) patients, mixed-cell in 23 (41.8%) and lymphocyte-rich in 4 (7.3%) patients. According to the Hodgkin’s lymphoma staging, 13 (23.6%), 18 (32.7%), 19 (34.5%) and 5 (9.1%) patients were classified as having stages I, II, III, and IV, respectively.ABVD, COPP, and MOPP/ABVD hybrid protocols were applied to 49 (89.1%), 2 (3.6%), and 4 (7.3%) patients, respectively. Three and 6 cycles of chemotherapy were given to 50.9% and 49.1% of all patients, respectively. Out of the 55 patients in our study, 50 (90.9%) received radiotherapy, 42 (76.4%) of whom underwent neck/mantle radiotherapy. Twenty-two (52.4%), 14 (33.3%), and 6 (14.3%) patients received a total dose of 2520 cGy, 3060 cGy, and 3600 cGy neck/mantle radiotherapy, respectively. Twenty-two (52.4%) out of 42 patients who received neck/mantle radiotherapy also received radiotherapy to other regions, while the remaining 20 (47.6%) patients did not. Statistical analysis revealed no differences with regard to thyroid function among the different histopathological groups, nor among the various stage or chemotherapy groups. There were also no differences detected among patients who were exposed to 3 or to 6 cycles of treatment. Exposure or non-exposure to radiotherapy or to radiotherapy to the neck/mantle region also did not appear to lead to differences in thyroid function. On the other hand, statistically significant differences were found regarding thyroid dysfunction among patients receiving different doses of neck/mantle radiotherapy. Thyroid function was normal in 72.7%, 92.9%, and 16.7% of patients who received a total dose of 2520 cGy, 3060 cGy, and 3600 cGy neck/mantle radiotherapy, respectively. However, binary comparisons of the three doses revealed statistically significant differences between the 2520 cGy dose and the 3600 cGy dose (p=0.022) and also between the 3060 cGy dose and 3600 cGy dose (p=0.002). Subclinical and overt hypothyroidism was diagnosed in 11 (78.6%) and 3 (21.4%) patients with abnormal thyroid function tests, respectively. Table 3 lists the features in individual patients with abnormal thyroid function tests. Thyroid dysfunction was detected after the first, second, third, fourth and fifth year of treatment in 3, 2, 3, 4 and 2 patients, respectively. No statistically significant correlation was found between time passed after treatment and thyroid dysfunction. 

**Table 1:**
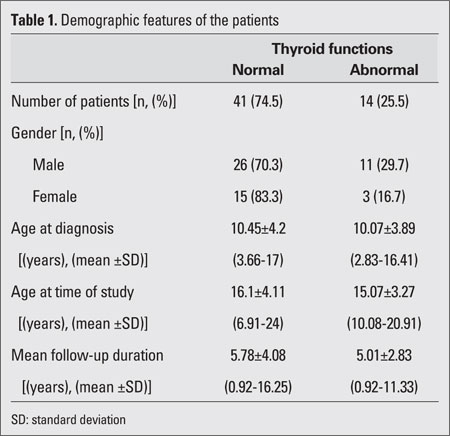
Demographic features of the patients

**Table 2:**
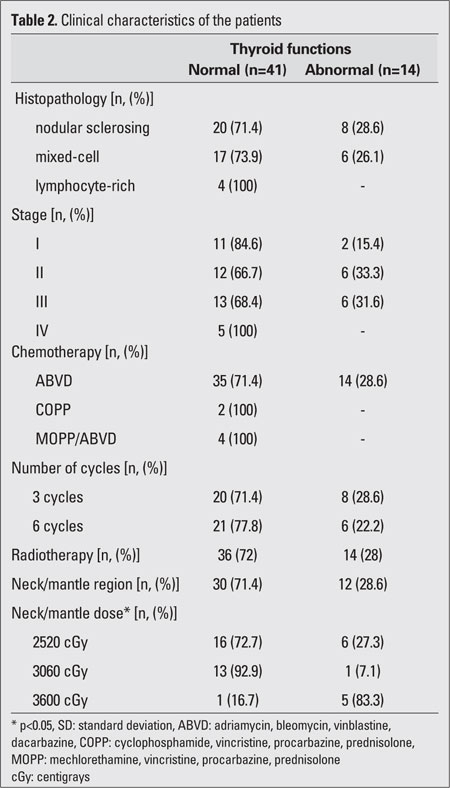
Clinical characteristics of the patients

**Table 3:**
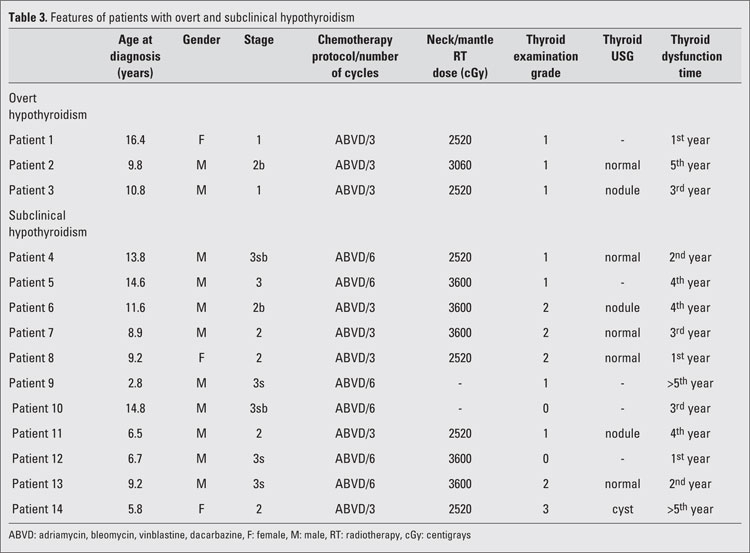
Features of patients with overt and subclinical hypothyroidism

## DISCUSSION

**Discussion**

In parallel to enhanced survival rates of pediatric Hodgkin’s lymphoma patients by current chemotherapy and radiotherapy protocols, an increase in early and late complication rates was also noted (14,15,16). The risk of thyroid disorder development was found to be 52% and 67% after 20 and 26 years of survival, respectively, in a study including 1787 patients with Hodgkin’s lymphoma (14). We found a thyroid disorder rate of 25.5% within a mean follow-up duration of 5.54 years in our study group of 55 patients, all in complete remission following chemotherapy and radiotherapy. Of the 14 patients who showed abnormal thyroid function, 78.6% were diagnosed as subclinical and 11.4% as overt hypothyroidism. Subclinical hypothyroidism following exposure to radiotherapy has been reported to have a high incidence in Hodgkin’s patients (17). Hunger et al (18) detected subclinical chemical hypothyroidism in 9 (16%) out of 57 patients who received a combination of chemotherapy and low-dose radiotherapy in their study with a mean follow-up duration of 6.7 years. In another study (19), subclinical hypothyroidism was found in 22 (63%) out of 35 patients with Hodgkin’s lymphoma who received mantle radiotherapy; some of these patients reportedly recovered within time. Periodical thyroid function tests revealed altered levels of at least one thyroid hormone. Elevation in TSH level was the most commonly encountered disorder. In one study (20), a high TSH was reported in 29 (50%) out of 58 patients who had received chemotherapy and low-dose (15-25 Gy) mantle radiotherapy. In our study, we also observed a significant correlation between thyroid dysfunction and dose of the neck/mantle radiotherapy. 

Radiotherapy dose, which was 44-45 Gy previously, was reduced to 15-25.5 Gy due to late complications (1). In a study (21) on 119 Hodgkin’s lymphoma patients, TSH levels were found to be elevated in 17% and 78% of those who received low-dose (lower than 2600 rad) and high-dose (higher than 2600 rad) mantle radiotherapy to the neck, respectively. Since thyroid dysfunction occurred 18 and 31 months after low- and high-dose radiotherapy, respectively, it is generally stated that it takes a time period of 3 to 5 years for thyroid function to be affected. In another study (6) with a mean follow-up duration of 30 years, thyroid dysfunction, the most common (17.1%) of which was hypothyroidism (p=0.0001), was detected in 34% of 1791 Hodgkin’s lymphoma patients who received chemotherapy and radiotherapy; the risk of hypothyroidism was increased by high-dose radiotherapy, advanced diagnosis age and female gender. Hyperthyroidism was detected in 5% of these patients - an 8-fold increased risk compared with controls (p=0.0001). The risk of thyroid nodule development was 27-fold that of controls after a mean latent period of 14 years in this same study and the risk of nodule development was correlated with high dose radiotherapy (higher than 2500 cGy) and female gender. 7% of the nodules were of malignant nature. In addition, thyroid cancer was found in 20 survivors in that study, which is 18-fold of that expected in a community. In a study (14) on 1677 patients with Hodgkin’s lymphoma who received radiotherapy to the thyroid gland region, at least one nodule was detected in 2.6% of them. The risk of thyroid nodule development was found to be increased 27-fold in a controlled study after a mean latent period of 14 years. 7% of the nodules were of malignant nature.  Independent risk factors were reported as female gender and high dose (higher than 25 Gy) of radiotherapy (6). The risk of benign or malignant thyroid disease development subsequent to chemotherapy and radiotherapy is reported to be increased 1.74- or 36.4-fold, respectively. The length of duration after treatment and the age at which the treatment was initiated increase this risk. Although radiotherapy is significantly correlated with such risks, the risk caused by chemotherapy is not clear. However, it is generally accepted that a combination of chemotherapy and radiotherapy further enhances the risk of benign or malignant thyroid disease (22). A study (23) on 1380 patients with Hodgkin’s lymphoma reported thyroid neoplasm in 19 (1.4%) patients within a mean follow-up period of 15.3 years (range: 4.2-29.7 years). Radiotherapy, chemotherapy and a combination of radiotherapy plus chemotherapy were applied to 5 (26%), 1 (5%) and 13 (69%) patients, respectively. Cumulative incidence for secondary malignancy was calculated as 10.6% and 26.3% on the 20th and 30th years, respectively. In another study (24) conducted between 1960 and 1990, thyroid malignancies were detected in 14 (1.5%) out of 930 patients with Hodgkin’s lymphoma within a mean follow-up period of 14.4 (range: 8.5-23) years. Another study (25) reported that the overall cumulative 10- and 20-year second malignancy rates were 2.45% and 7.29%, respectively, in 358 pediatric Hodgkin’s lymphoma patients, and that only one patient who received chemotherapy alone developed thyroid malignancy on the 13th year after treatment. Acharya et al (12) reported that 18 out of 33 patients with pediatric Hodgkin’s lymphoma developed thyroid neoplasm after a mean mantle radiotherapy dose of 2400 cGy (range: 1000-4200 cGy) to the neck within a mean period of 13 (range 6.2-30.1) years and that 39% of those neoplasms were of malignant nature (13 papillary carcinoma and 2 follicular carcinoma). Another study (26) found that 10% of 67 patients developed thyroid nodules of malignant nature 16.2 years after completion of radiotherapy with an average 35 Gy dose. In our study, we detected thyroid nodules in 4 (7.3%) patients within an average follow-up period of 5.5±3.7 years. One of these patients received surgery and the nodule was reported to be benign in nature. One reason why we did not encounter malignant thyroid nodules in our patients might be the relatively short duration of follow-up after completion of treatment. In conclusion, our study shows that thyroid dysfunction occurs in approximately one-fourth of pediatric Hodgkin’s lymphoma patients within an average period of 5.5 years after completion of treatment. Our findings also demonstrate that thyroid functions can be affected as early as the first year after completion of treatment. Thyroid dysfunction was found to statistically correlate with the dose of neck/mantle radiotherapy. The reason why the rate of thyroid dysfunction in our study was smaller than those in other reports could be that we applied a lower dose as mantle radiotherapy. It is also known that the rate of detection of thyroid nodules increases with duration of follow-up. Thus, periodic evaluation of the thyroid gland in Hodgkin’s lymphoma patients who received radiotherapy and chemotherapy needs to be started at an early period after treatment and continued for many years. 

**Conflict of Interest**

No author of this paper has a conflict of interest, including specific financial interests, relationships, and/or affiliations relevant to the subject matter or materials included in this manuscript.
